# Diverse functional connectivity patterns of resting-state brain networks associated with good and poor hand outcomes following stroke

**DOI:** 10.1016/j.nicl.2019.102065

**Published:** 2019-11-20

**Authors:** Wenjun Hong, Qixiang Lin, Zaixu Cui, Feiwen Liu, Rong Xu, Chaozheng Tang

**Affiliations:** a Department of Rehabilitation Medicine, Nanjing Drum Tower Hospital, The Affiliated Hospital of Nanjing University Medical School, Nanjing, China; b Capacity Building and Continuing Education Center, National Health Commission of the People’s Republic of China, Beijing, China; c Department of Neurology, School of Medicine, Emory University, Atlanta, USA; d Department of Psychiatry, Perelman School of Medicine, University of Pennsylvania, Philadelphia, USA; e Department of Rehabilitation Medicine, Huashan Hospital, Fudan University, Shanghai, China; f State Key Laboratory of Cognitive Neuroscience and Learning, Beijing Normal University, Beijing, China; g Department of Rehabilitation Medicine, Chengdu Second People's Hospital, Chengdu, China

**Keywords:** Functional connectivity, Independent component analysis, ICA, Resting-state functional magnetic resonance imaging, Functional brain network, Stroke, PPH, partially paralyzed hand, CPH, completely paralyzed hand, resting-state fMRI, resting-state functional magnetic resonance imaging, FC, functional connectivity, ICA, independent component analysis, FMA-HW, Fugl-Meyer Assessment of Hand and Wrist, MN, motor network, SMN, sensorimotor network, CN, cerebellum network, DMN, default mode network, ECN, executive control network, FPN, frontoparietal network, DAN, dorsal attention network, AN, auditory network, VN, visual network.

## Abstract

Motor stroke has been characterized by disruptions in multiple large-scale functional brain networks. However, it remains unclear whether stroke patients with good hand outcomes show different connectivity profiles within and between networks from those with poor hand outcomes. In this cross-sectional study, we recruited 52 chronic subcortical stroke patients [illness duration (mean ± SD): 16 ± 16.2 months] and 52 healthy controls from the local hospital and community from June 2010 to August 2016. We first performed independent component analysis (ICA) on resting-state fMRI data to extract fifteen resting-state networks. Then, we compared the functional connectivity within and between networks across 52 healthy controls, 26 patients with a partially paralyzed hand (PPH), and 26 patients with a completely paralyzed hand (CPH). Compared to the patients with a PPH, the patients with a CPH showed increased connectivity in the contralesional sensorimotor cortex within the contralesional sensorimotor network; the increased connectivity was negatively correlated with the performance of the paretic hand. Moreover, the patients with a CPH, compared to those with a PPH, showed decreased strengths of connectivity between the ipsilesional sensorimotor network and both the dorsal sensorimotor network and ventral visual network; the decreased strengths of connectivity were positively correlated with the performance of the paretic hand. Collectively, our findings suggest that stroke patients with different hand outcomes show distinct functional reorganization patterns in large-scale brain networks. These findings shed light on the network-level neuromechanisms that help explain why stroke survivors in the chronic stage show different hand outcomes.

## Highlights

• Stroke patients with good and poor hand outcomes show different connectivity patterns.
• Disrupted functional network connectivity is associated with hand outcomes.
• The findings may motivate the development of noninvasive, targeted brain stimulation.

## 1 Introduction

Stroke patients suffering from hand paresis typically have difficulties in daily activities (Veerbeek et al., 2011). Although brain reorganization drives spontaneous recovery following stroke (Grefkes and Fink, 2014; Loubinoux et al., 2003; Rehme et al., 2011b), this recovery is usually incomplete and can vary considerably across individuals (Stinear, 2010). Most functional recovery following stroke occurred within the first three months (Ramsey et al., 2017), while the neural processes and behavioral improvements still showed mild plasticity at the chronic stage (Rehme et al., 2012). Despite the fact that stroke frequently damages subcortical regions (Corbetta et al., 2015), empirical evidence regarding functional reorganization in chronic subcortical stroke patients with disparate hand outcomes is still scarce. Our previous studies have demonstrated that stroke patients suffering from partial hand paresis and complete hand paresis showed different neuroplasticity patterns in topological organization (Yin et al., 2014) and in frequency-dependent local spontaneous oscillations (Zhao et al., 2018a). These studies indicated that it is critical to consider the impact of the hand deficits on functional reorganization in chronic stroke, which may be helpful for understanding the neurophysiologic mechanisms of different hand outcomes after chronic stroke and motivating the development of noninvasive, targeted brain stimulation (Grefkes and Fink, 2012; Grefkes et al., 2010; Koch and Hummel, 2017).

Resting-state functional magnetic resonance imaging (fMRI) (Biswal et al., 1995; Fox and Raichle, 2007) has been considered an attractive technique for mapping neuroplasticity in a lesioned brain (Carter et al., 2010; Lu et al., 2011; Wang et al., 2010). With resting-state fMRI, it has been demonstrated that motor stroke involves not only reorganization of sensorimotor networks but also alterations in functional connectivity (FC) with higher cognitive control areas (e.g., the middle frontal gyrus) (Park et al., 2011) and primary perception areas (e.g., the calcarine gyrus) (Tang et al., 2016). Moreover, several FC studies have revealed potential neural mechanisms of spontaneous and rehabilitation-driven motor recovery after stroke (Park et al., 2011; Rehme et al., 2011a; Volz et al., 2016). Motor execution requires functional interactions between distributed but related brain regions (Zhao et al., 2018b), and focal lesions may disrupt remote cortical networks and result in impaired functional processes (Siegel et al., 2016; Wang et al., 2014). However, to date, the nature and relevance of abnormal network reorganization for different hand outcomes after chronic subcortical stroke remain poorly understood.

Independent component analysis (ICA) is a powerful data-driven method for investigating the intrinsic functional architecture of large-scale brain networks (Damoiseaux et al., 2006; Smith et al., 2012) and allows an unbiased exploration of the association between brain networks and neuropsychiatric disorders (Greicius et al., 2004; Zhou et al., 2010). Resting-state functional networks derived from ICA have nearly 40% genetic heritability (Glahn et al., 2010), an electrophysiological basis (Brookes et al., 2011; de Pasquale et al., 2010; He et al., 2008; Mantini et al., 2007), frequency-specific characteristics (Vidaurre et al., 2018), underlying structural pathways (Greicius et al., 2009), and similarities to the known spatial patterns of brain activation induced by domain-specific tasks (Smith et al., 2009). In addition, previous studies have found that resting-state functional networks were spatiotemporally overlapping (Karahanoglu and Van De Ville, 2015) and highly reproducible and reliable for mapping the human brain (Damoiseaux et al., 2006; Zuo et al., 2010). Disrupted functional network connectivity has been revealed in several neuropsychiatric disorders, such as chronic pain (Malinen et al., 2010), nicotine dependence (Lerman et al., 2014), attention-deficit/hyperactivity disorder (Kessler et al., 2014), and depression (Kaiser et al., 2015), and throughout the whole course of Alzheimer's disease (Filippini et al., 2009; Greicius et al., 2004; Jones et al., 2016; Seeley et al., 2009; Sorg et al., 2007); these disruptions were usually accompanied by abnormalities in the default mode, executive control, and salience networks. Using the ICA approach, one study found that well-recovered stroke patients showed disrupted connectivity within and between networks in the sensory processing, dorsal attention, frontoparietal, and default mode networks (Wang et al., 2014). Another study found that stroke patients with severe to mild motor deficits showed abnormal connectivity in more widespread networks, such as the motor, visual, dorsal attention, executive control, and default mode networks (Zhao et al., 2018b). Furthermore, Zhao et al. found that the FC values in the ipsilesional inferior parietal lobule within the executive control network were negatively correlated with the paretic hand performance. However, this relationship was not found in Wang's study (Wang et al., 2014). To further clarify these inconsistent findings (Wang et al., 2014; Zhao et al., 2018b), the present study used a large and homogeneous sample with only left subcortical chronic stroke to systematically explore the functional reorganization patterns across 26 patients with a completely paralyzed hand (CPH), 26 patients with a partially paralyzed hand (PPH) and 52 healthy controls at the level of macroscopic brain networks.

Based on our previous studies among stroke subgroups (Yin et al., 2014; Zhao et al., 2018a) and the two studies that compared ICA networks between stroke patients and controls (Wang et al., 2014; Zhao et al., 2018b), we explored whether chronic stroke patients with a CPH would show more widespread FC reorganization within and between networks than those with a PPH, which might improve our pathophysiologic understanding of chronic stroke patients with different hand outcomes. Finally, we evaluated the associations between the performance of the paretic hand and the aberrant FC patterns within and between networks in chronic stroke patients.

## 2 Methods

### 2.1 Participants

This study was approved by the Ethics Review Board of East China Normal University and was conducted according to the guidelines of the Helsinki Declaration. Each participant was fully informed and signed a consent form before the study. A total of 107 participants [52 healthy controls, 28 CPH patients, and 27 PPH patients] were recruited in the present study. The inclusion criteria included (1) first-episode and left subcortical stroke; (2) age from 30 to 80 years; (3) disease duration ≥ 3 months; (4) pure motor deficits and Mini-Mental State Examination score ≥ 24; and (5) right handedness. The exclusion criteria included (1) any contraindication for MRI and (2) suffering from other neuropsychiatric disorders, severe atrial fibrillation and aphasia. The healthy controls were recruited from the nearby community through advertisements. All healthy controls had no neuropsychiatric history and cognitive complaints.

As described in our previous studies (Yin et al., 2014; Zhao et al., 2018a), the stroke patients were divided into PPH and CPH groups based on the Paralyzed Hand Function Assessment Scale (Supplementary Materials, Table A. 3). This scale involves assessment of five practical actions of the hand in daily life. The patients who could not complete any action were categorized as having CPH while those who could complete at least one of the five actions were categorized as having PPH.

### 2.2 Behavioral assessments

Before MRI data acquisition, we used the Fugl-Meyer Assessment of Hand and Wrist (FMA-HW) subscale to assess the performance of the paretic hand in chronic stroke patients. The FMA-HW subscale includes five wrist and seven hand items with a total possible score of 24 (Ramos-Murguialday et al., 2013; Zhao et al., 2018a), which was selected as the primary measurement. Additionally, the Mini-Mental State Examination was evaluated as a baseline-screening tool to ensure that the patients had pure motor deficits and sufficient cognitive ability to complete the study.

### 2.3 Data acquisition

Data were collected on a 3-Tesla SIEMENS Trio scanner. T1-weighted images were obtained using a magnetization prepared rapid gradient echo sequence: 192 sagittal slices, slice thickness = 1 mm, gap = =0.5 mm, repetition time (TR) = =1900 ms, echo time (TE) = =3.42 ms, inversion time (TI) = =900 ms, field of view (FOV) = =240 × 240 mm^2^, flip angle (FA) = =9°, and in-plane matrix size = =256 × 256. T2-weighted images were acquired using a turbo spin echo sequence: 30 axial slices, slice thickness = =5 mm, gap = =0 mm, TR = =6000 ms, TE = =93 ms, FOV = =220 × 220 mm^2^, FA = =120°, and in-plane matrix size = =320 × 320. Resting-state fMRI data were collected using an echo-planar imaging sequence: 30 axial slices, slice thickness = =4 mm, gap = =0.8 mm, TR = =2000 ms, TE = =30 ms, FOV = =220 × 220 mm^2^, FA = =90°, in-plane matrix size = =64 × 64, 240 vol, and an acquisition time that lasted for 8 min. Prior to fMRI scanning, all participants were instructed to keep their eyes closed, relax their mind, and not move as much as possible.

### 2.4 Lesion overlap analysis

**Fig. 1:**
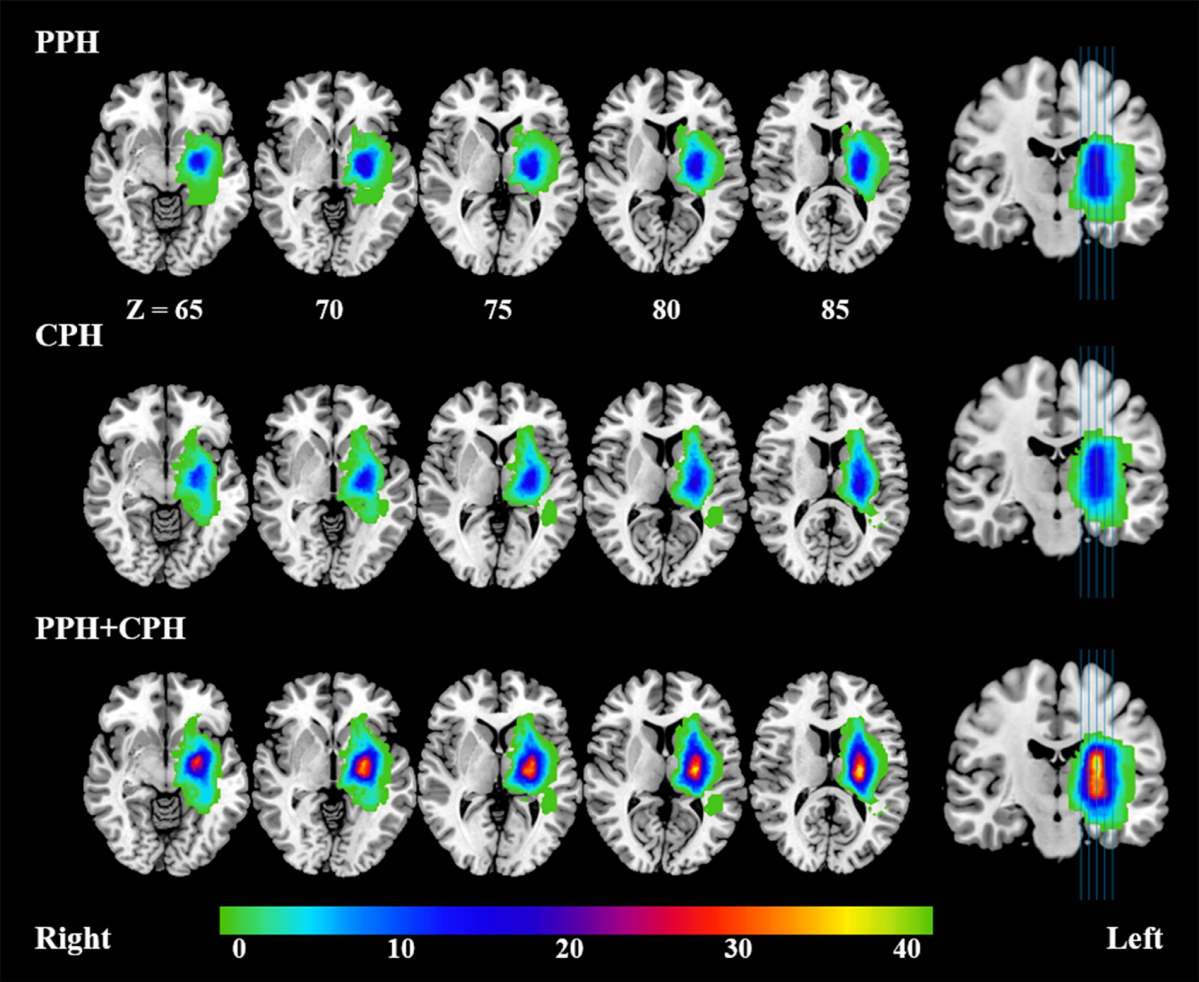
Lesion overlap map for the partially paralyzed hand (PPH) group, the completely paralyzed hand (CPH) group and all stroke patients. The color bar indicates the number of patients having lesions in each voxel. Left indicates the ipsilesional hemisphere.

Using MRIcron (https://www.nitrc.org/projects/mricron), the lesion profiles of each stroke patient were delineated on T2-weighted images by two physicians. Fig. 1 shows the lesion overlap map for the PPH patients, the CPH patients and all stroke patients.

### 2.5 Data preprocessing

Resting-state fMRI data were preprocessed using DPARSF within the DPABI (http://rfmri.org/DPARSF) (Yan et al., 2016). The procedure included (1) removal of the first 10 vol, (2) slice-timing correction, (3) realignment, (4) normalization to the MNI space with the lesion masks (Andersen et al., 2010) and unified segmentation of structural images (Ashburner and Friston, 2005), and (5) spatially smoothing (FWHM = =6 mm). We estimated the mean framewise displacement for each participant to characterize transient head motion (Jenkinson et al., 2002), and excluded the participants with a framewise displacement of more than 0.5 mm. Additionally, the participants with head motion exceeding 2 mm/degrees were removed. Finally, two patients with a CPH and one patient with a PPH were discarded. There were no significant differences in the framewise displacement between each pair of the three groups (Table 1).

**Table 1:** Demographics and clinical details of the participants.

	Controls (*n* = 52)	PPH group (*n* = 26)	CPH group (*n* = 26)	PPH vs Controls	CPH vs Controls	CPH vs PPH
	Mean ± SD	Mean ± SD	Mean ± SD	*p*-value	*p*-value	*p*-value
Age (years) ^a^	56 ± 8.23	56 ± 9.92	56 ± 10.22	0.80	0.58	0.89
Sex (male: female) ^b^	32 : 20	25 : 1	22 : 4	0.001	0.001	0.16
Hand dominance	right	right	right	–	–	–
Duration of illness (months) ^a^	–	16 ± 15.58	16 ± 17.36	–	–	0.87
Lesion volume (ml) ^a^	–	5.53 ± 4.89	7.77 ± 5.51	–	–	0.13
Mini-Mental State Examination ^a^	–	29 ± 1.36	29 ± 1.21	–	–	0.75
FMA-HW ^a^	–	12 ± 7.32	1.38 ± 1.20	–	–	**< 10^−9^**
Framewise displacement (mm) ^a^	0.09 ± 0.07	0.11 ± 0.05	0.15 ± 0.10	0.15	0.075	0.10

Note: ^a^ Independent *t*-test, ^b^ Chi-square test. CPH, completely paralyzed hand; PPH, partially paralyzed hand; FMA-HW, Fugl-Meyer Assessment of Hand and Wrist subscale.

### 2.6 Independent component analysis

**Fig. 2:**
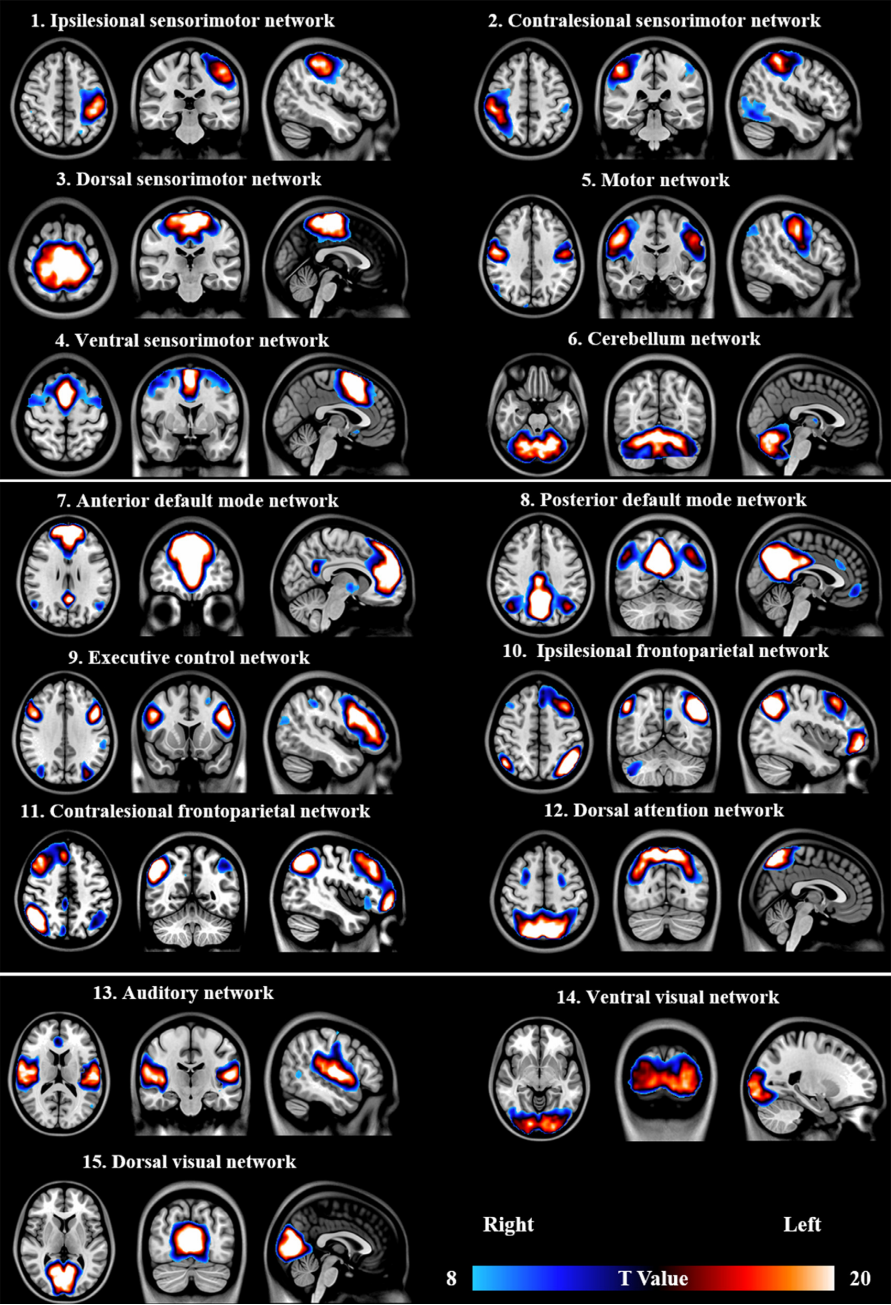
Fifteen resting-state brain networks were identified using independent component analysis. The color bar represents the T values ranging from 8 to 20. Left indicates the ipsilesional hemisphere.

The ICA was conducted using GIFT (http://mialab.mrn.org/software/gift/). First, the preprocessed data were automatically decomposed into 40 independent components using a 3-step principal component analysis. Second, the group independent components were evaluated using the Infomax algorithm. Finally, the robust group ICA3 method (Erhardt et al., 2011) was applied to back-reconstruct the individual spatial maps and time courses. Then, the individual maps of each brain network were transformed to z scores (Fisher's r-to-z transformation) and entered into a random effect one-sample *t*-test (*T* > 8) to obtain the group-level network maps in SPM12 (https://www.fil.ion.ucl.ac.uk/spm). We projected the functional networks onto MNI space for visualization using MRIcron. Consistent with previous studies (Damoiseaux et al., 2006; Smith et al., 2009; Zuo et al., 2010), fifteen brain networks were visually identified (Fig. 2): the motor network (MN), ipsilesional/contralesional/dorsal/ventral sensorimotor network (SMN), cerebellum network (CN), anterior/posterior default mode network (DMN), executive control network (ECN), ipsilesional/contralesional frontoparietal network (FPN), dorsal attention network (DAN), auditory network (AN), and ventral/dorsal visual network (VN).

### 2.7 Functional connectivity analysis within networks

To determine the group differences in within-network FC, comparisons among the three groups were conducted using one-way analyses of variance. We used the AlphaSim method with a corrected *p* < 0.0001 (voxel-wise *p* < 0.001, cluster size = =23 voxels) to generate the F-map according the Monte Carlo simulation (10,000 simulations, FWHM = =5.6 mm, with a gray matter mask). We further conducted *post hoc* two-sample *t*-tests for the brain networks showing significant group effects. The effects of age, sex, and framewise displacement were adjusted for all of these analyses. Multiple comparisons of the *post hoc* t-tests were corrected using a false discovery rate method (FDR, *q* < 0.01).

### 2.8 Functional connectivity analysis between networks

We first extracted the time series of the 15 networks for each participant from the ICA and calculated the Pearson's correlation coefficients of the time series of each pair of the 15 networks. Then, Fisher's r-to-z transformation was conducted for r values. To determine the group differences in between-network FC, comparisons among the three groups were conducted using one-way analyses of variance (*q* < 0.05, FDR corrected). We further conducted *post hoc* two-sample t-tests for between-network FC showing significant group effects (*q* < 0.05, FDR corrected).

### 2.9 Exploring brain-behavior correlations

Pearson's correlation analysis was conducted to detect the relationship between the FC indices of the surviving network patterns and the FMA-HW scores across all stroke patients (*n* = 52). Specifically, regarding the within-network FC, the brain areas that displayed significant FC differences between the CPH and PPH groups were selected. Then, the averaged z scores of the FC among all voxels within these brain areas were extracted and correlated with the FMA-HW scores across all stroke patients. Regarding the between-network FC, the functional network connectivity with significant differences between the CPH and PPH groups was extracted and correlated with the FMA-HW scores across all stroke patients. A statistical threshold of *p* < 0.025 was adopted (Bonferroni corrected).

## 3 Results

### 3.1 Demographic and clinical data

Fifty-five stroke patients and 52 healthy controls were recruited. Two patients with a CPH and one patient with a PPH were discarded during the data preprocessing because of excessive head motion. Finally, 52 patients (i.e., 26 PPH and 26 CPH) and 52 controls were included. The details of all patient lesions are shown in Supplementary Materials (Fig. A. 1). As expected, the FMA-HW scores of PPH patients were significantly higher than those of CPH patients (two-sample *t*-test, *p* < 10^−9^). Furthermore, there were no significant differences in age, handedness dominance, duration of illness, lesion volume, Mini-Mental State Examination score and framewise displacement among the groups, with the exception of gender between the PPH group and controls (chi-square test, *p* = 0.001), and between the CPH group and controls (chi-square test, *p* = 0.001) (Table 1).

### 3.2 Disrupted functional connectivity within networks in the three groups

**Fig. 3:**
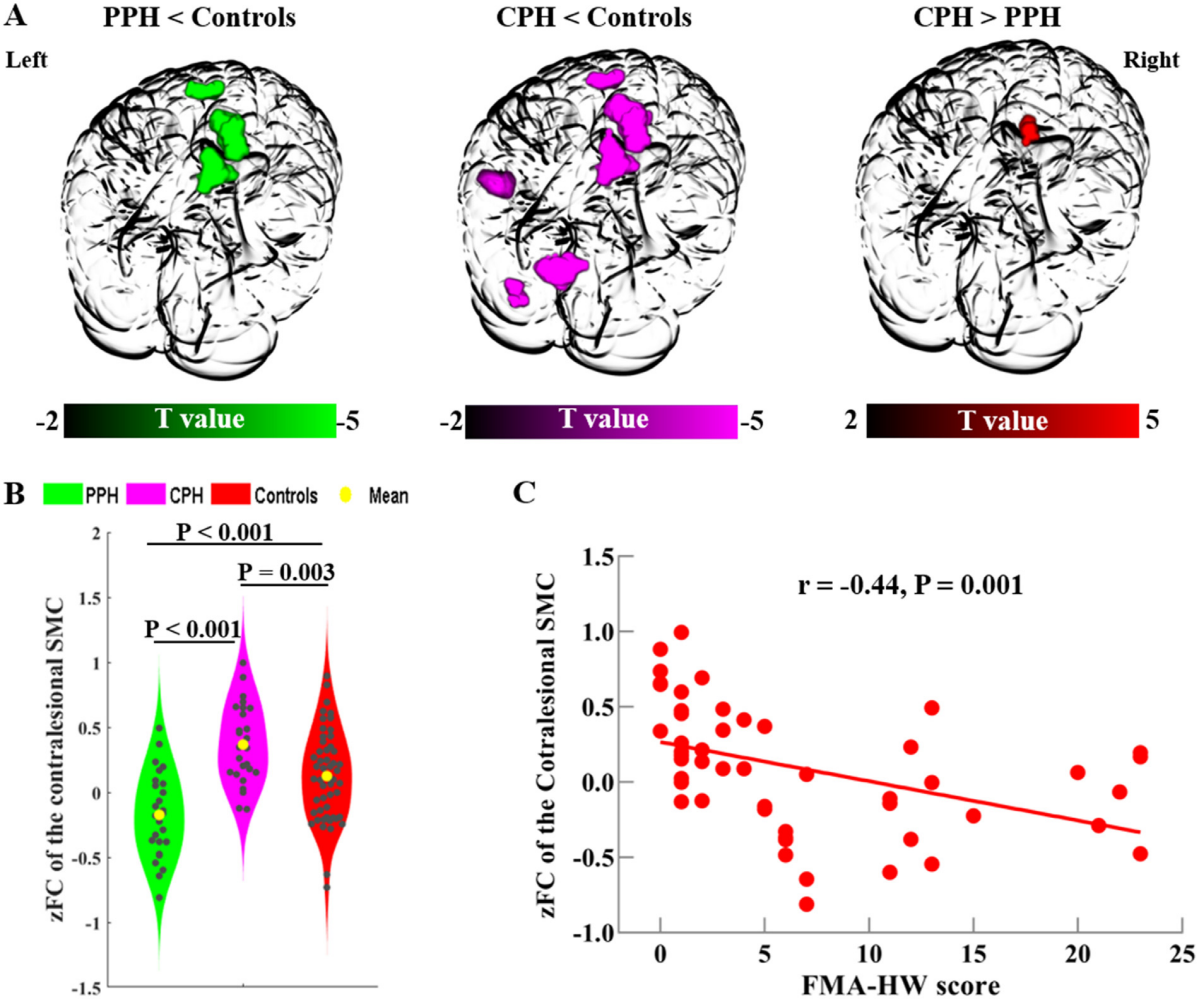
Disrupted functional connectivity within networks among the three groups. (A) Disrupted within-network FC patterns between each pair of the PPH, CPH and controls, which are rendered in the 3D images (*q* < 0.01, FDR corrected). (B) The violin plot displays the intergroup differences in the zFC values in the contralesional SMC within the contralesional sensorimotor network among the PPH, CPH and controls. (C) The zFC values of the contralesional SMC are negatively correlated with the FMA-HW scores across all stroke patients (Bonferroni corrected). The color bars represent the T values from the intergroup comparisons. CPH, completely paralyzed hand; PPH, partially paralyzed hand; FMA-HW, Fugl-Meyer Assessment of Hand and Wrist; FC, functional connectivity; SMC, sensorimotor cortex. Left indicates the ipsilesional hemisphere.

Regarding within-network FC, we found significant group differences among the three groups in the contralesional sensorimotor cortex within the contralesional SMN, the contralesional precentral gyrus and superior parietal lobe within the dorsal SMN, the ipsilesional supplementary motor area within the ventral SMN, the ipsilesional superior temporal gyrus within the AN, the ipsilesional middle occipital gyrus within the ventral VN, and the contralesional calcarine within the dorsal VN (Supplementary Materials, Fig. A. 3). *Post hoc* comparisons revealed that FC in both the PPH and CPH groups significantly decreased in the contralesional precentral gyrus and superior parietal lobe within the dorsal SMN and the ipsilesional supplementary motor area within the ventral SMN compared to FC in the controls. Compared to the controls, the CPH group also showed decreased FC in the ipsilesional superior temporal gyrus within the AN, the ipsilesional middle occipital gyrus within the ventral VN, and the contralesional calcarine within the dorsal VN. Most importantly, compared to the PPH patients, the CPH patients showed increased FC in the contralesional sensorimotor cortex within the contralesional SMN (Fig. 3A and Supplementary Materials, Table A. 1; *q* < 0.01, FDR corrected). Finally, we found that the zFC values of the contralesional sensorimotor cortex in the CPH patients were higher than those in the PPH patients and controls (Fig. 3B).

### 3.3 Disrupted functional connectivity between networks in the three groups

**Fig. 4:**
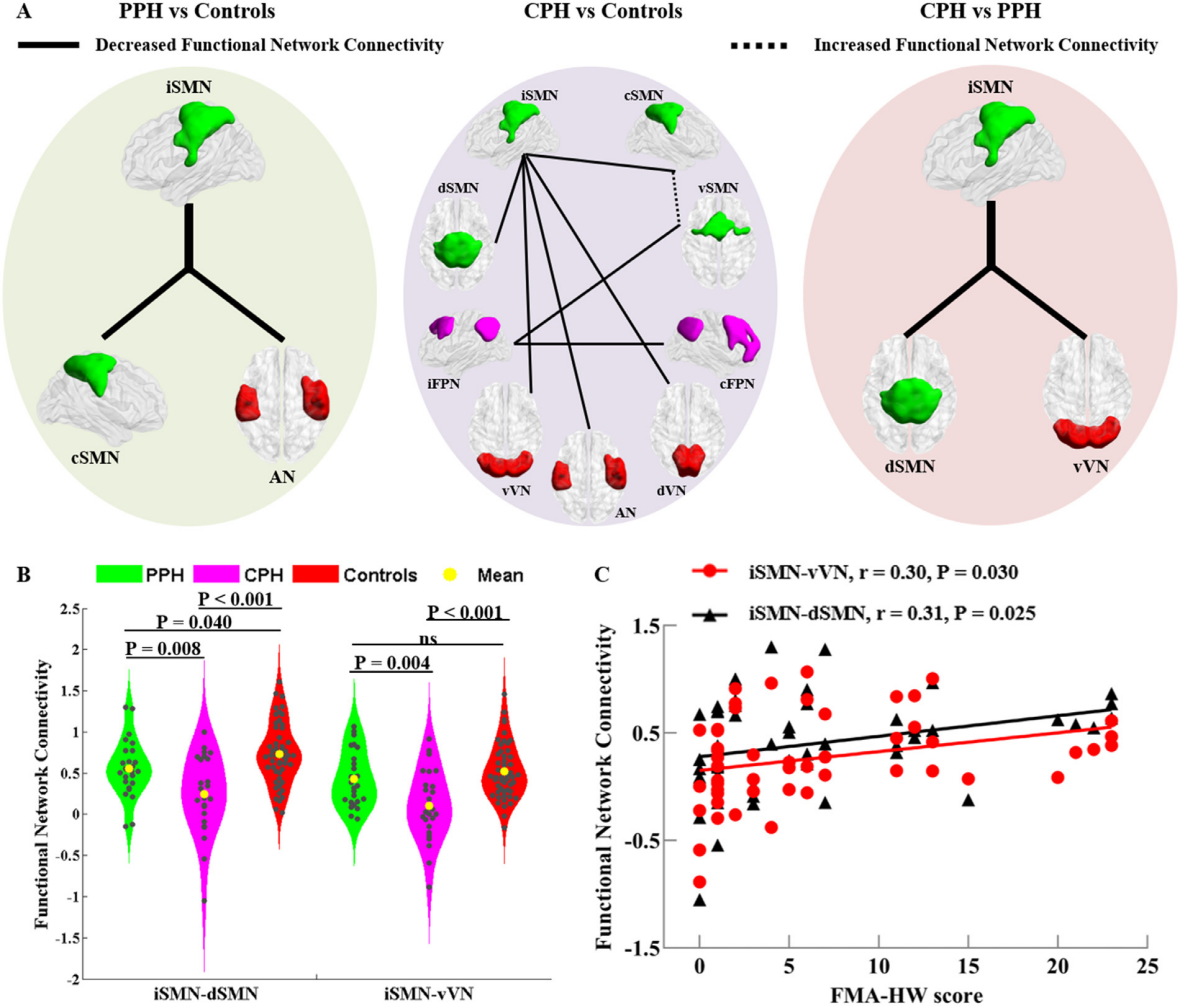
Disrupted functional connectivity between networks among the three groups. (A) Diverse functional network connectivity patterns between each pair of the PPH, CPH and controls. The solid and dotted lines represent decreased and increased between-network connectivity, respectively (*q* < 0.05, FDR corrected). (B) The violin plot displays the intergroup differences in connectivity between the iSMN and the vVN and between the iSMN and the dSMN among the PPH, CPH and controls. (C) The functional network connectivity of the iSMN with the vVN and the iSMN with the dSMN are positively correlated with the FMA-HW scores across all stroke patients (Bonferroni corrected, except for the correlation between iSMN-vVN connectivity and FMA-HW scores.). CPH, completely paralyzed hand; PPH, partially paralyzed hand; i/c/d/v SMN, ipsilesional/contralesional/dorsal/ventral sensorimotor network; i/c FPN, ipsilesional/contralesional frontoparietal network; AN, auditory network; v/d VN, ventral/dorsal visual network; FMA-HW, Fugl-Meyer Assessment of Hand and Wrist; ns, not significant. Left indicates the ipsilesional hemisphere. BrainNet Viewer was used for 3D surface visualization (www.nitrc.org/projects/bnv) (Xia et al., 2013).

Regarding between-network FC, we found significant group differences among the three groups between the ipsilesional SMN and the contralesional SMN, dorsal SMN, AN, ventral VN, and dorsal VN, between the ventral SMN and the contralesional SMN and ipsilesional FPN, and between the ipsilesional FPN and contralesional FPN (Supplementary Materials, Fig. A. 4). *Post hoc* comparisons revealed that both the PPH and CPH groups showed decreased FC between the ipsilesional SMN and the contralesional SMN and AN compared to the controls. Meanwhile, compared to the controls, the CPH group showed widespread decreased FC between the ipsilesional SMN and the dorsal SMN, ventral VN, and dorsal VN and between the ipsilesional FPN and the ventral SMN and contralesional FPN. Moreover, compared to the controls, the CPH group showed increased FC between the contralesional SMN and the ventral SMN. Most importantly, we found decreased FC between the ipsilesional SMN and both the dorsal SMN and ventral VN in the CPH group compared to the PPH group (Fig. 4A and Supplementary Materials, Table A. 2; *q* < 0.05, FDR corrected). Finally, we found that FC between the ipsilesional SMN and both the dorsal SMN and ventral VN in the CPH patients was lower than that in the PPH patients and controls (Fig. 4B).

### 3.4 Functional connectivity correlated with paretic hand performance

At the voxel level, we found that mean zFC in the contralesional sensorimotor cortex within the contralesional SMN was negatively correlated with the FMA-HW scores across all stroke patients (*r* = =−0.44, *p* = 0.001) (Fig. 3C). Additionally, we replicated the relationship between zFC within the contralesional SMN and FMA-HW scores in a voxel-wise manner (Supplementary Materials, Fig. A. 2), which was consistent with our main finding. At the network level, FC between the ipsilesional SMN and the dorsal SMN and between the ipsilesional SMN and the ventral VN were positively correlated with the FMA-HW scores (*r* = 0.31, *p* = 0.025 and *r* = 0.30, *p* = 0.030, respectively) (Fig. 4C). All the correlations between FC and behavior survived the Bonferroni correction, except for the correlation between the ipsilesional SMN-ventral VN and FMA-HW scores.

## 4 Discussion

The present study evaluated the distinct FC patterns within and between functional networks in chronic stroke patients with different hand outcomes. Regarding the within-network reorganization, we found that FC in the contralesional sensorimotor cortex within the contralesional SMN was significantly increased in the CPH patients compared to the PPH patients. Regarding the between-network reorganization, we found that FC between the ipsilesional SMN and both the dorsal SMN and ventral VN was significantly decreased in the CHP patients compared to the PPH patients. More importantly, we found that the disrupted FC patterns in large-scale brain networks were associated with paretic hand performance in left subcortical chronic stroke patients. These findings provide new insights into the neurophysiologic mechanisms underlying the different hand outcomes following chronic stroke, which may motivate the development of noninvasive, targeted brain stimulation.

### 4.1 Disrupted functional connectivity within networks

Compared to healthy controls, stroke patients with complete recovery had disrupted within-network FC in the cognitive, executive control, and primary perception networks (Wang et al., 2014), while those with mild to severe motor deficits had decreased within-network FC in the precentral gyrus within the MN (Zhao et al., 2018b). Consistent with the findings from Zhao et al. (2018b), we found that both the CPH and PPH patients, compared with the controls, showed decreased within-network FC in the contralesional precentral gyrus within the dorsal SMN. Furthermore, compared to the controls, both the CPH and PPH patients showed decreased within-network FC in the contralesional superior parietal lobe and the ipsilesional supplementary motor area, while the CPH patients also showed widespread decreases in within-network FC in the perception areas. These results were not found in the previous two studies (Wang et al., 2014; Zhao et al., 2018b), and the discrepancies between the two ICA studies and our present study could have been caused by differences in patient characteristics, such as different motor deficits, lesion sides, and stroke types. Therefore, our study clarified these inconsistent findings by showing that the differences in within-network FC between chronic stroke patients and controls were modulated by the degree of motor deficits (Zhao et al., 2018a).

For poorly recovered stroke patients, the contralesional sensorimotor cortex possibly contributes to the functional outcomes of the ipsilateral hand (Netz et al., 1997). In this regard, several studies have indicated that sustained dominant activation of the contralesional sensorimotor cortex impeded the functional recovery of the paretic hand (Calautti et al., 2007; Johansen-Berg et al., 2002; Rehme et al., 2011b). Investigators have found increased functional recruitment of the contralesional premotor cortex in chronic stroke patients with a greater severity of motor deficits (Johansen-Berg et al., 2002), particularly in acute stroke patients with higher-level deficits (Rehme et al., 2011b). Other similar findings also suggested that hyperactivation in the contralesional sensorimotor cortex was related to poor performance in chronic stroke patients (Calautti et al., 2007). Consistent with these findings (Calautti et al., 2007; Johansen-Berg et al., 2002; Rehme et al., 2011b), we found that the CPH patients showed increased within-network FC in the contralesional sensorimotor cortex compared to the PPH patients, indicating that the severe hand deficits of the CPH patients were accompanied by excessive recruitment of contralesional sensorimotor resources. Moreover, the mean zFC in the contralesional sensorimotor cortex was negatively correlated with the FMA-HW scores across all stroke patients, suggesting that greater recruitment of the contralesional sensorimotor cortex is an undesirable indicator in the chronic stage of stroke patients. Additionally, empirical studies of repetitive transcranial magnetic stimulation have shown the benefits of inhibiting the contralesional hemisphere on motor performance (Mansur et al., 2005; Takeuchi et al., 2005). Thus, our findings indicate that the contralesional sensorimotor cortex may have played a crucial role in chronic stroke patients who suffered different hand outcomes (Rehme et al., 2011b) and might be considered an alternative target for promoting hand rehabilitation after chronic stroke.

### 4.2 Disrupted functional connectivity between networks

Large-scale functional reorganization across networks in stroke patients reported in two previous studies were inconsistent (Wang et al., 2014; Zhao et al., 2018b). For instance, Zhao et al. found that stroke patients, compared to healthy controls, showed decreased connectivity between the ipsilesional FPN and the MN and DAN, between the contralesional FPN and the ECN (but increased connectivity with the DMN), and between the posterior DMN and the VN (Zhao et al., 2018b). However, Wang et al. found that stroke patients, compared to healthy controls, showed decreased connectivity between the VN and the AN and between the posterior DMN and the FPN (Wang et al., 2014). The FPN is a lateralized network that supports the function of high-order cognitive control (Nomura et al., 2010). In the present study, compared with the controls, the CPH patients showed decreased connectivity between the ipsilesional FPN and the ventral SMN and contralesional FPN. The decreased connectivity between the ipsilesional FPN and the ventral SMN supports the previous finding that the impaired cognitive control of the ipsilesional FPN to SMN may disrupt motor control in chronic severe stroke patients (Zhao et al., 2018b). Moreover, compared with the controls, the CPH patients showed widespread decreased connectivity between the ipsilesional SMN and the somatomotor systems (contralesional SMN and dorsal SMN) and primary perception systems (AN, ventral VN and dorsal VN) and increased connectivity between the contralesional SMN and the ventral SMN. We speculated that the impaired large-scale functional interactions among the SMN, FPN, AN, and VN may have contributed to the poor hand outcomes in the CPH patients, while increased connectivity between the contralesional SMN and the ventral SMN may represent a compensatory mechanism.

Previous studies have revealed that sensorimotor recovery was correlated with both interhemispheric FC (Carter et al., 2010) and the normalization of network configurations in the bilateral sensorimotor cortex (van Meer et al., 2012; Volz et al., 2016). In the present study, compared with the controls, both the CPH and PPH patients showed decreased connectivity between the ipsilesional SMN and the contralesional SMN, which indicated that the disrupted interhemispheric interactions between the bilateral SMN may contribute to motor deficits observed in chronic stroke patients (Carter et al., 2010). Compared to the PPH patients, the CPH patients showed decreased connectivity between the ipsilesional SMN and both the dorsal SMN and ventral VN. Moreover, the connectivity between the ipsilesional SMN and the dorsal SMN and between the ipsilesional SMN and the ventral VN (not significant after Bonferroni correction) were positively correlated with the FMA-HW scores. These findings suggest that sensory information from proprioception and vision may play crucial roles in guiding and adjusting top-down motor control (Mattingley et al., 1998), which deepens our understanding of visuomotor coordination for hand dysfunction substrates. Collectively, our study further extends previous research (Wang et al., 2014; Zhao et al., 2018b) regarding the existence of large-scale functional brain network reorganization in chronic stroke patients with different hand outcomes, and this may be helpful for clarifying inconsistent findings in previous studies.

### 4.3 Limitations and perspectives

Five limitations in the present study should be noted. First, due to the protective role of female hormones for stroke incidence and the increased vulnerability of males to left hemisphere stroke, there is a heavy male predominance in our stroke samples (Gibson and Attwood, 2016). To mitigate this problem, we controlled for gender as a nuisance covariate in our statistical analysis. Second, a large slice thickness (4 mm) parameter was used in the data acquisition, which inevitably reduced the spatial resolution of the functional imaging. Future studies using the recently developed multiband/multiplexed echo-planar imaging methods (Feinberg et al., 2010; Moeller et al., 2010) could enhance the quality of fMRI data with unprecedented sampling rates for full-brain coverage. Third, all data presented here were cross-sectional, which precludes the prediction of hand outcomes. Future studies could use longitudinal follow-up data to predict the prognosis of either CPH or PPH in stroke patients by using brain network biomarkers. Fourth, the patients in the present study all suffered from left subcortical stroke, and it would be helpful to recruit patients with right subcortical stroke to verify the generalizability of our findings. Finally, previous studies have suggested that the lesion load of the corticospinal tract may serve as an imaging biomarker for predicting the motor deficits of the upper extremity in stroke patients (Carter et al., 2012; Feng et al., 2015; Zhu et al., 2010). Thus, it is a promising field to explore the relationship of corticospinal tract lesion load, functional reorganization, and motor deficits by using a mediation model.

## 5 Conclusions

We found that the neuroplasticity in left subcortical chronic stroke patients with different hand outcomes mainly occurred in the multiple sensorimotor and primary perception subnetworks, and these large-scale functional reorganization profiles may contribute to the understanding of the pathophysiologic mechanisms of preserved hand ability in chronic stroke patients. Furthermore, the contralesional sensorimotor area identified in this study may serve as an alternative target to motivate the development of noninvasive brain stimulation (e.g., transcranial magnetic stimulation) in chronic stroke patients.

## Funding

This work was supported by the Natural Science Foundation of Zhejiang Province [grant number LGF19H270001] and Key Project of Medical Science and Technology Development Foundation [grant number ZKX18012, Nanjing Department of Health].

## Declaration of Competing Interest

None.

## Appendix Supplementary materials

Image, application 1
