# Supplementary material for: Diverse functional connectivity patterns of resting-state brain networks associated with good and poor hand outcomes following stroke
Source: Neuroimage Clin. 2019 Nov 20;24:102065. doi: 10.1016/j.nicl.2019.102065 (PMC6889370; doi:10.1016/j.nicl.2019.102065)
Supplement: Supplementary file 1 [file mmc1.docx]

**Fig. A. 1.** The lesion location of each PPH and CPH patient is shown in T2-weighted axial images. Left indicates the ipsilesional hemisphere.


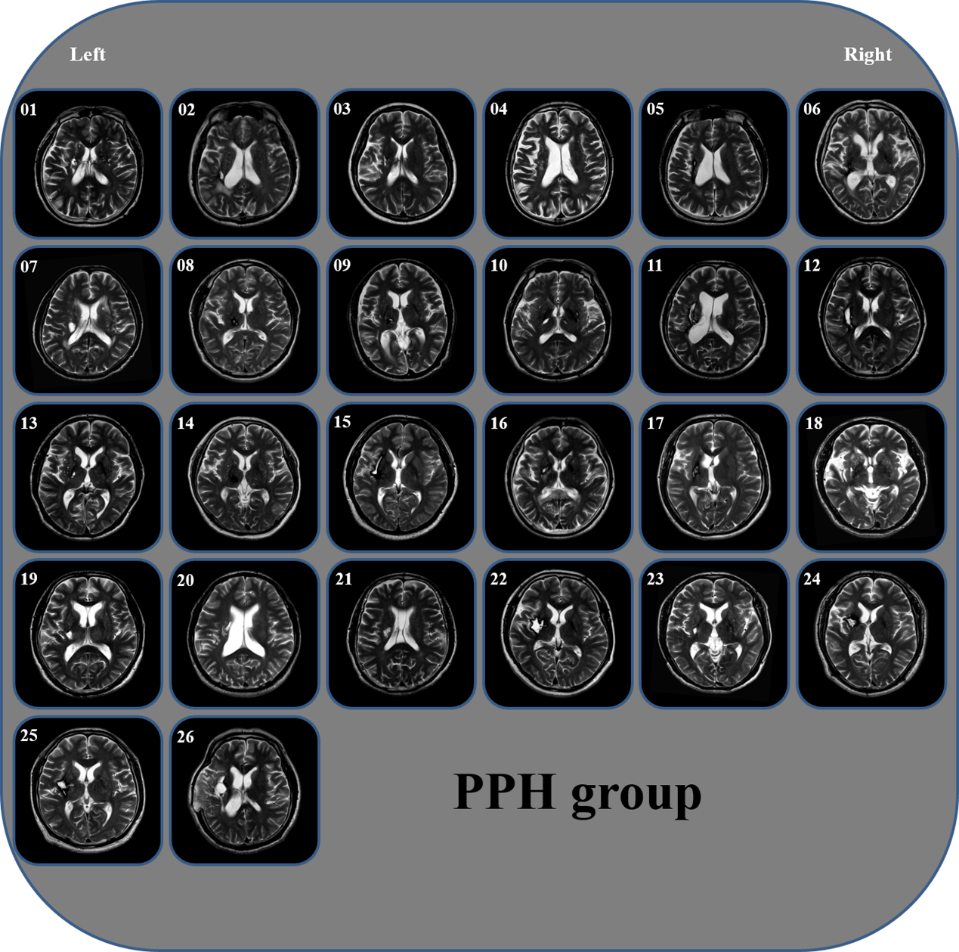


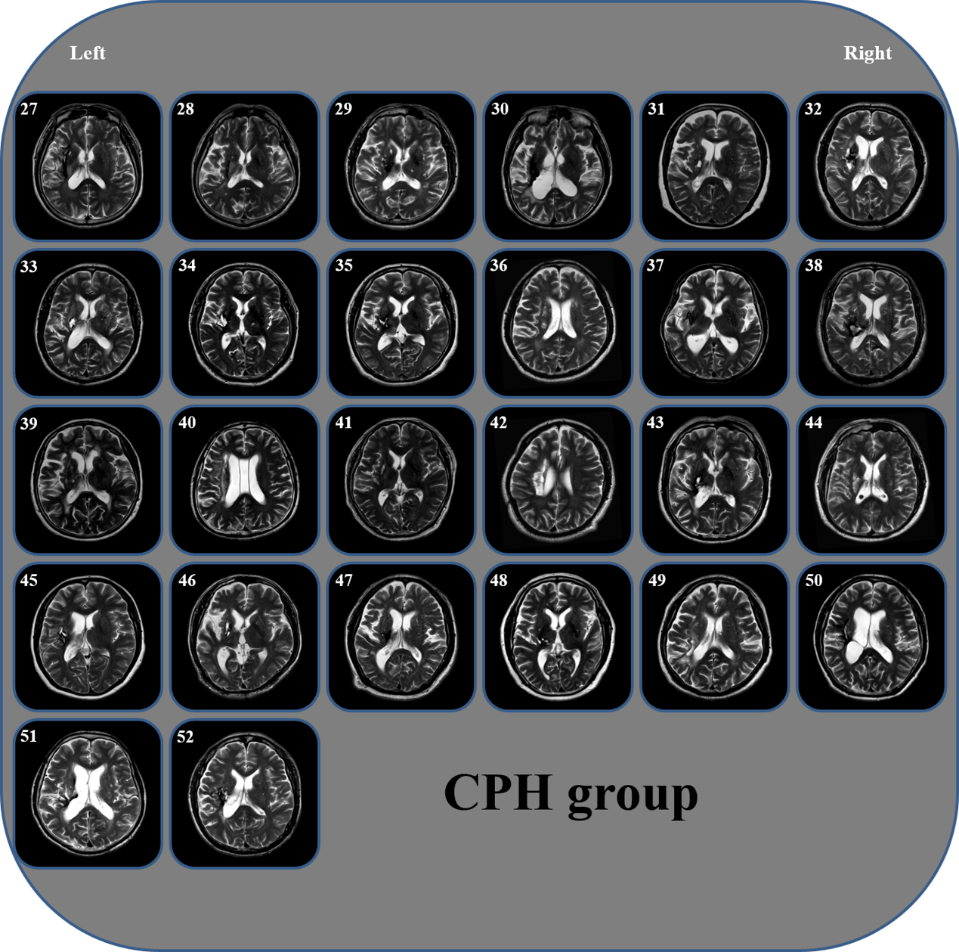


**Fig. A. 2.** Correlation result between zFC within the contralesional SMN and FMA-HW scores across all stroke patients. (A) represents the map of the correlation coefficient for the surviving cluster and (B) represents the map of log(*p*) (*p* < 0.001 and cluster > 5 voxels, uncorrected).


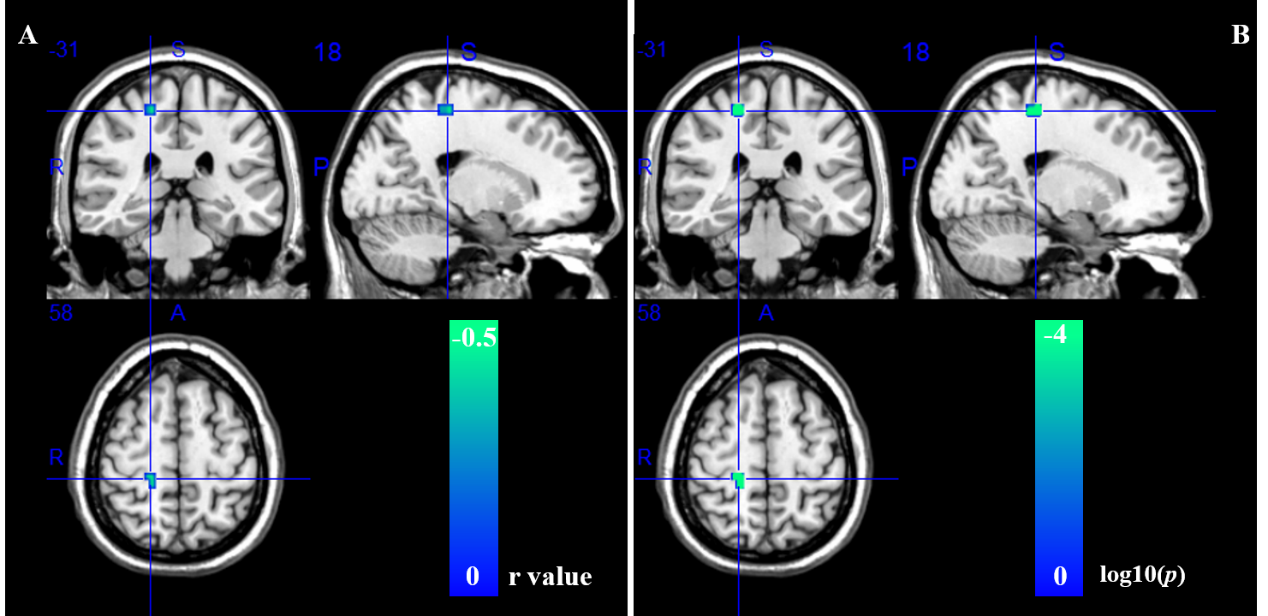


**Fig. A. 3.** The significant group effects of within-network FC among the three groups were evaluated using one way analyses of variance.

**
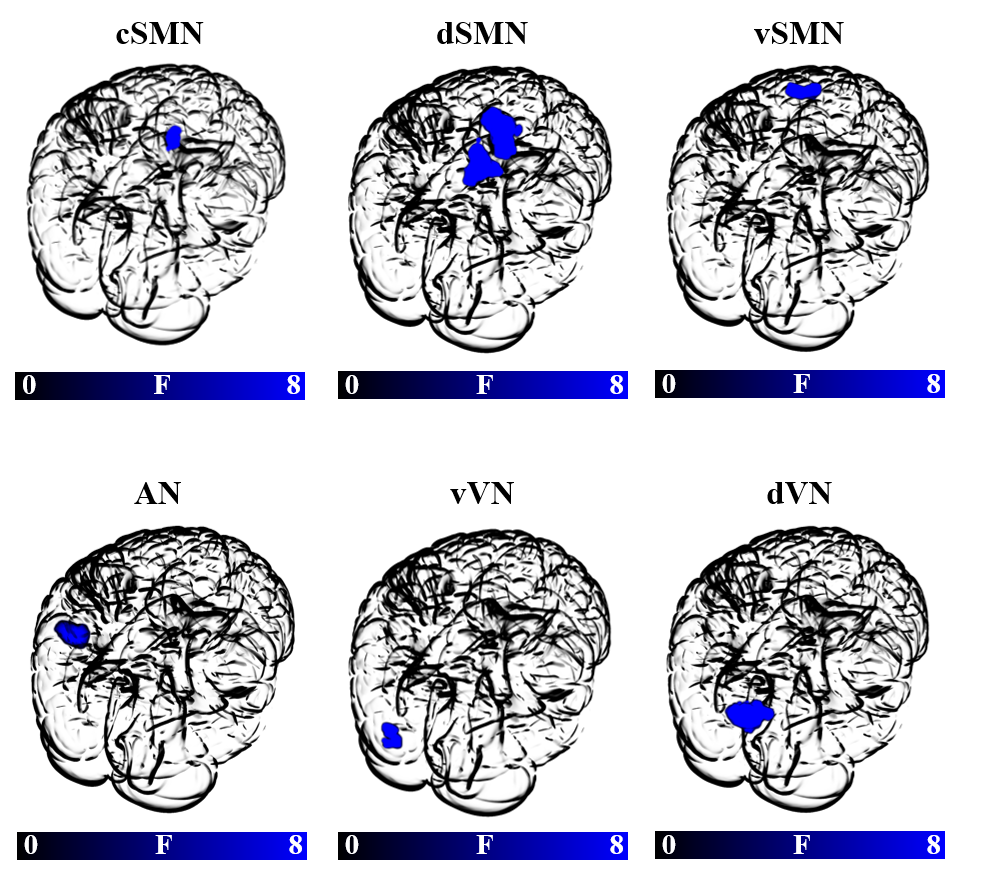
**

**Fig. A. 4.** The significant group effects of between-network FC among the three groups were evaluated using one way analyses of variance.

**
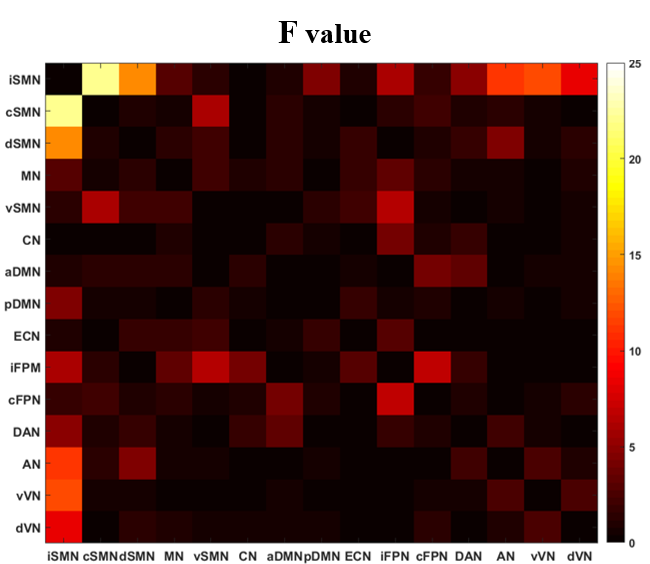
**

**
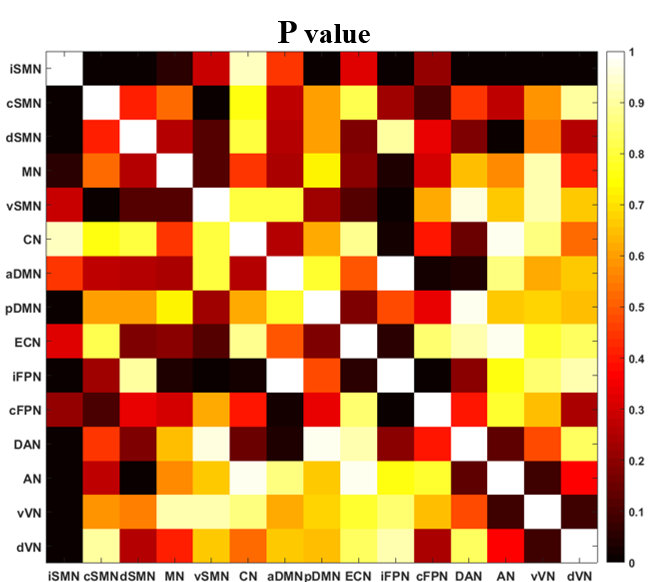
**

**Table A. 1.** Disrupted within-network connectivity between each pair of the three groups.

| **Regions** | **Networks** | **MNI** | | | **Cluster** | **T** |
| --- | --- | --- | --- | --- | --- | --- |
|  |  | **X** | **Y** | **Z** |  |  |
| **PPH < Controls** | | | | | | |
| Contralesional precentral gyrus | Dorsal SMN | 15 | -21 | 69 | 124 | -4.58 |
| Contralesional superior parietal lobe | Dorsal SMN | 18 | -51 | 69 | 95 | -4.69 |
| Ipsilesional supplementary motor area | Ventral SMN | -12 | -6 | 72 | 38 | -4.79 |
| **CPH < Controls** | | | | | | |
| Contralesional precentral gyrus | Dorsal SMN | 6 | -18 | 72 | 145 | -6.07 |
| Contralesional superior parietal lobe | Dorsal SMN | 18 | -60 | 63 | 98 | -5.58 |
| Ipsilesional supplementary motor area | Ventral SMN | -6 | 0 | 69 | 37 | -4.36 |
| Ipsilesional superior temporal gyrus | AN | -57 | -30 | 12 | 57 | -6.30 |
| Ipsilesional middle occipital gyrus | Ventral VN | -21 | -93 | 0 | 37 | -4.60 |
| Contralesional calcarine | Dorsal VN | 3 | -78 | 9 | 100 | -5.86 |
| **CPH > PPH** | | | | | | |
| Contralesional sensorimotor cortex | Contralesional SMN | 18 | -27 | 60 | 26 | 5.45 |

**Note:** CPH, completed paralyzed hand; PPH, partially paralyzed hand; SMN, sensorimotor network; MN, motor network; FPN, frontoparietal network; AN, attention network; VN, visual network. Statistical threshold: *q* < 0.01 (FDR corrected).

**Table A. 2.** Disrupted between-network connectivity between each pair of the three groups.

| **Surviving between network connectivity** | **T value** | **P value** |
| --- | --- | --- |
| **PPH < Controls** | | |
| ipsilesional SMN with contralesional SMN | -4.11 | < 0.0001 |
| ipsilesional SMN with AN | -2.71 | 0.0071 |
| **CPH < Controls** | | |
| ipsilesional SMN with contralesional SMN | -6.16 | < 0.0001 |
| ipsilesional SMN with dorsal SMN | -5.01 | < 0.0001 |
| ipsilesional SMN with ventral VN | -4.75 | < 0.0001 |
| ipsilesional SMN with dorsal VN | -4.12 | < 0.0001 |
| ipsilesional SMN with AN | -3.85 | 0.0002 |
| ipsilesional FPN with ventral SMN | -3.41 | 0.0010 |
| ipsilesional FPN with contralesional FPN | -4.11 | < 0.0001 |
| **CPH > Controls** | | |
| contralesional SMN with ventral SMN | 4.34 | < 0.0001 |
| **CPH < PPH** | | |
| ipsilesional SMN with ventral VN | -3.05 | 0.0036 |
| ipsilesional SMN with dorsal SMN | -2.74 | 0.0084 |

**Note:** CPH, completed paralyzed hand; PPH, partially paralyzed hand; SMN, sensorimotor network; MN, motor network; FPN, frontoparietal network; AN, attention network; VN, visual network. Statistical threshold: *q* < 0.05 (FDR corrected).

**Table A. 3.** Paralyzed Hand Function Assessment Scale.

| **Action items** | **Illustration** | **Hand function classification** | **Evaluation criteria** |
| --- | --- | --- | --- |
| 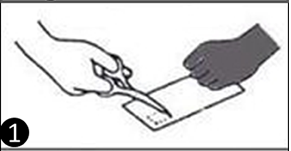 | The affected hand stabilizes a piece of paper on the table, and the unaffected hand uses a shear to cut the paper. | Disabled hand | Could not complete any activities. |
| 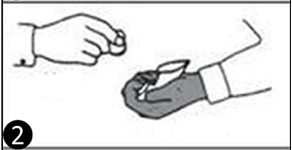 | The affected hand holds a wallet, and the unaffected hand takes a coin from the wallet. | Assistant hand C | Finished one of the five activities. |
| 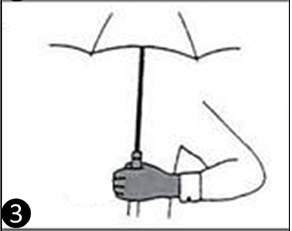 | The affected hand holds an unfolded umbrella in the air for at least 10 s. | Assistant hand B | Finished two of the five activities. |
|  |  | Assistant hand A | Finished three of the five activities. |
| 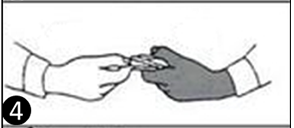 | The affected hand controls a nail scissor to trim nails of the unaffected hand. | Practical hand B | Finished four of the five activities. |
| 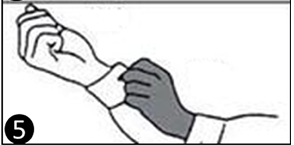 | The affected hand buttons the cuff of the unaffected side. | Practical hand A | Completed all the five activities. |

**Note.** The darker hand denotes the affected hand.
